# Sub-differentiation of PI-RADS 3 lesions in TZ by advanced diffusion-weighted imaging to aid the biopsy decision process

**DOI:** 10.3389/fonc.2023.1092073

**Published:** 2023-02-10

**Authors:** Kun-Peng Zhou, Hua-Bin Huang, Chao Bu, Zhong-Xing Luo, Wen-Sheng Huang, Li-Zhi Xie, Qing-Yu Liu, Jie Bian

**Affiliations:** ^1^ Seventh Affiliated Hospital, Sun Yat-sen University, Shenzhen, China; ^2^ GE Healthcare, Beijing, China; ^3^ Radiology, Second Affiliated Hospital of Dalian Medical University, Dalian, Liaoning, China

**Keywords:** prostate cancer, PI-RADS, intravoxel incoherent motion, stretched exponential model, diffusion kurtosis imaging

## Abstract

**Background:**

Performing biopsy for intermediate lesions with PI-RADS 3 has always been controversial. Moreover, it is difficult to differentiate prostate cancer (PCa) and benign prostatic hyperplasia (BPH) nodules in PI-RADS 3 lesions by conventional scans, especially for transition zone (TZ) lesions. The purpose of this study is sub-differentiation of transition zone (TZ) PI-RADS 3 lesions using intravoxel incoherent motion (IVIM), stretched exponential model, and diffusion kurtosis imaging (DKI) to aid the biopsy decision process.

**Methods:**

A total of 198 TZ PI-RADS 3 lesions were included. 149 lesions were BPH, while 49 lesions were PCa, including 37 non-clinical significant PCa (non-csPCa) lesions and 12 clinical significant PCa (csPCa) lesions. Binary logistic regression analysis was used to examine which parameters could predict PCa in TZ PI-RADS 3 lesions. The ROC curve was used to test diagnostic efficiency in distinguishing PCa from TZ PI-RADS 3 lesions, while one-way ANOVA analysis was used to examine which parameters were statistically significant among BPH, non-csPCa and csPCa.

**Results:**

The logistic model was statistically significant (χ2 = 181.410, *p*<0.001) and could correctly classify 89.39% of the subjects. Parameters of fractional anisotropy (FA) (*p=0.004*), mean diffusion (MD) (*p=0.005*), mean kurtosis (MK) (*p=0.015*), diffusion coefficient (D) (*p=0.001*), and distribute diffusion coefficient (DDC) (*p=0.038*) were statistically significant in the model. ROC analysis showed that AUC was 0.9197 (CI 95%: 0.8736-0.9659). Sensitivity, specificity, positive predictive value and negative predictive value were 92.1%, 80.4%, 93.9% and 75.5%, respectively. FA and MK of csPCa were higher than those of non-csPCa (all *p*<0.05), while MD, ADC, D, and DDC of csPCa were lower than those of non-csPCa (all *p*<0.05).

**Conclusion:**

FA, MD, MK, D, and DDC can predict PCa in TZ PI-RADS 3 lesions and inform the decision-making process of whether or not to perform a biopsy. Moreover, FA, MD, MK, D, DDC, and ADC may have ability to identify csPCa and non-csPCa in TZ PI-RADS 3 lesions.

## Introduction

The pathological and clinical features of prostate cancer (PCa) vary in different anatomical regions of the prostate. Compared to peripheral zone (PZ) cancers, transition zone (TZ) cancers have lower Gleason scores, higher tumor volumes, higher prostate-specific antigen (PSA) levels, and are often confined to the prostate (1). Although patients with TZ cancers have a more favorable prognosis than patients with PZ cancers, TZ cancers are more difficult to detect, especially those with small tumor volume (1, 2).

Multiparametric MRI has been increasingly used to detect and locate tumors in patients highly suspected of PCa based on clinical examination results, such as PSA level and digital rectal exam. The recently updated version 2.1 of Prostate Imaging-Reporting and Data System (PI-RADS) guidelines have standardized the imaging acquisition and interpretation, providing more detailed principles for evaluating prostate lesions to reduce interreader variability (3).

Generally, PI-RADS 1 or 2 lesions are considered as “negative” MRI, and have a negative predictive value of more than 90% for clinically significant prostate cancer (csPCa, Gleason≥3+4) (4, 5). In addition, the PI-RADS guidelines do not recommend biopsy for PI-RADS 1 or 2 lesions. On the other hand, prostate lesions with PI-RADS 4 or 5 are considered PCa, and targeted biopsy is inevitable. Yet, performing a biopsy for these PI-RADS 3 lesions (intermediate lesions) is still a matter of debate (6, 7). For PI-RADS 3 lesions, several studies have found that about 6.5%-60% were confirmed as PCa by biopsy, while 4.1%-21% were csPCa (7–9). Gosein et al. (10) reported that 27.3% of PI-RADS 3 lesions located in TZ were PCa, among which 9.1% were csPCa.

Several studies have shown that csPCa in PI-RADS 3 have higher PSA density, lower prostate volume, and apparent diffusion coefficient (ADC) value compared with benign lesions in PI-RADS 3 (11–14). Using conventional scanning sequences makes it difficult to distinguish TZ cancers from fibromuscular (stromal) benign prostatic hyperplasia (BPH) as both of these conditions can manifest low T2 signal intensity on MR images. Hansen et al. (15) have found that PI-RADS 3 lesions with low ADC value located in anterior of TZ, as well as the irregular shape, ill-defined border, and homogenous T2 signal intensity can predict the PCa in TZ; however, only ill-defined border and low ADC value help predict scPCa. We found that the evaluation criteria for PI-RADS 3 lesions in Hansen’s study were PI-RADS Version 1 and Version 2. Tamada et al. (16) found that compared with PI-RADS Version 2.1, PI-RADS Version 2 could classify some PI-RADS 4 or 5 lesions as PI-RADS 3 lesions. Advanced diffusion-weighted imaging (DWI) such as intravoxel incoherent motion (IVIM), stretched exponential model, and diffusion kurtosis imaging (DKI) can reflect microstructural complexity of tumor tissues, what’s more, DKI can provide information about non-Gaussian diffusion. some studies have demonstrated that parameters derived from IVIM, stretched exponential model, and DKI was useful in the detection and assessment of PCa aggressiveness and may even be superior to DWI (17–19).

In strict compliance with the evaluation criteria of PI-RADS V2.1, the present study attempted to differentiate PCa and BPH in PI-RADS 3 lesions in TZ with advanced diffusion-weighted imaging (IVIM, DKI, stretched exponential model) and to analyze which parameters have potential value in predicting csPCa.

## Materials and methods

### Patients

**Figure 1:**
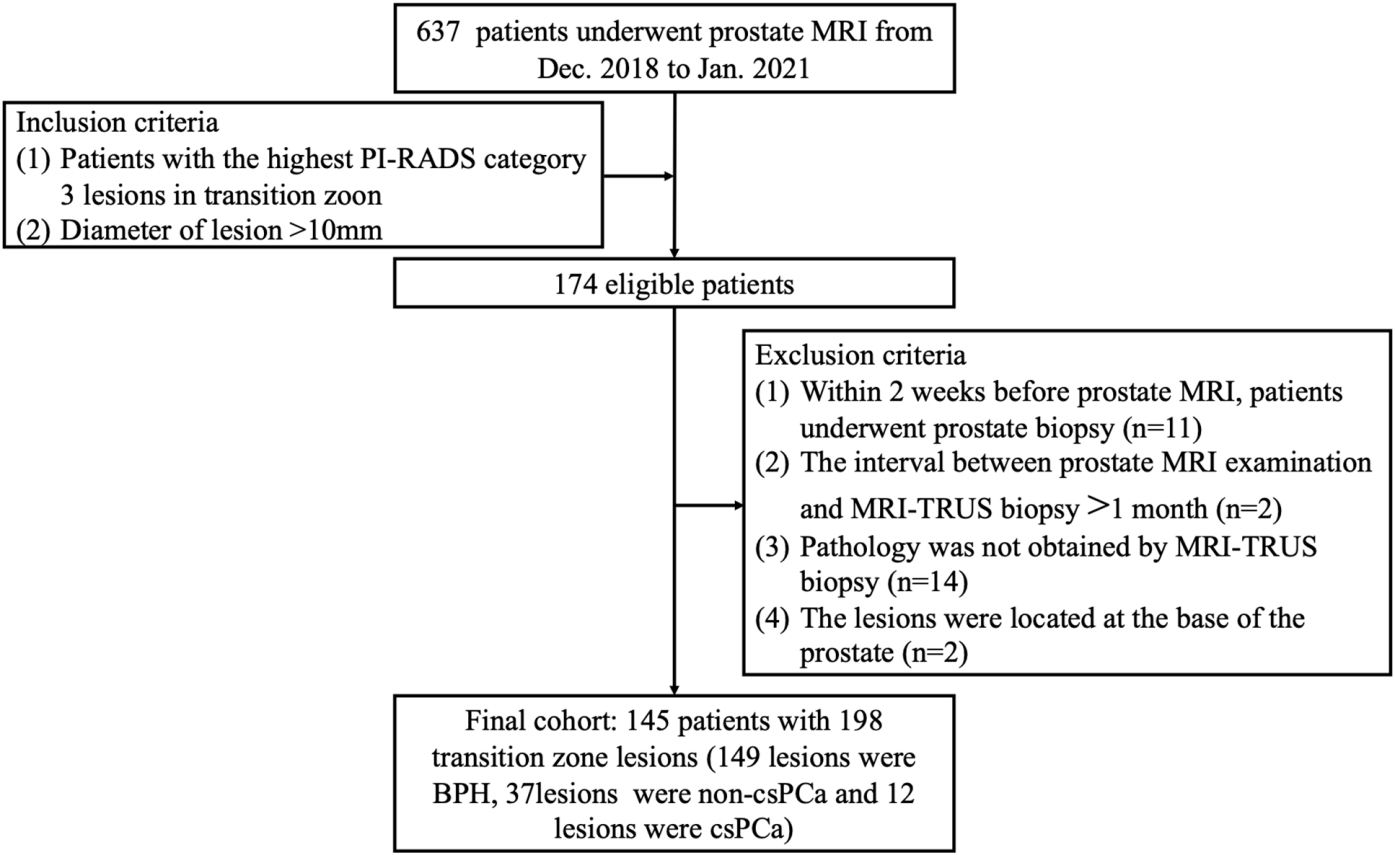
Flow diagram of the study population.

This study was approved by the ethics committee (subheading on ethics committee incompleted). A total of 637 patients with an abnormal increase in serum PSA (T-PSA>10 ng/mL; T-PSA, 4-10 ng/mL and F-PSA/T-PSA<0.25) underwent prostate MRI examination between December 2018 and January 2021. Inclusion criteria: patients with the highest PI-RADS category 3 lesions in TZ with a diameter larger than 10 mm. Exclusion criteria (1): patients underwent prostate biopsy (n=11) within 2 weeks before prostate MRI (2); the interval between prostate MRI examination and magnetic resonance imaging-Transrectal ultrasound (MRI-TRUS) biopsy >1 month (n=2) (3); pathology was not obtained by MRI-TRUS fusion biopsy (n=14) (4); the lesions were located at the base of the prostate so that biopsy specimens could not be obtained (n=2). Finally, 145 patients with 198 PI-RADS category 3 lesions located in TZ were included in the present study. Among 198 PI-RADS 3 lesions in TZ, 149 lesions were pathologically confirmed as BPH, while 49 lesions as PCa, among which 37 were non-clinically significant prostate cancer (non-cs PCa, Gleason 3 + 3) and 12 were csPCa (**Figure 1**).

### MRI techniques

All the enrolled patients underwent prostate MRI examination at 3.0 T MRI (GE Discovery MR 750W) with an 8-channel phased-array coil. Conventional prostate MR images, including axial T1-weighted imaging, axial T2-weighted imaging, coronal T2-weighted imaging, sagittal T2-weighted imaging, diffusion-weighted imaging, and dynamic contrast-enhanced imaging, were obtained according to the scanning protocol recommended by PI-RADS version 2.1. DKI was obtained with the following parameters: repetition time msec/echo time msec, 3800–4600/87.4; section thickness, 3 mm; intersection gap, 0.5 mm; field of view, 28 cm×28 cm; matrix, 128×128; 15 directions and 3 *b* values (0, 1000 and 2000 s/mm^2^) were used. IVIM were obtained with following parameters: repetition time msec/echo time msec, 3500–4400/87.5; section thickness, 3.6 mm; intersection gap, 0.5 mm; field of view, 28 cm×28 cm; matrix, 128×128; and 11 *b* values (0, 25, 50, 75, 100, 150, 200, 400, 800, 1200 and 2000 s/mm^2^) were used. The details of MRI scan sequences and parameters are shown in **Table 1**.

**Table 1:** Acquisition parameters of the multiparametic MRI protocol.

Sequence	TR/TE (ms)	Slice/Gap (mm)	FOV (cm^2^)	Matrix	b (s/mm^2^)	Direction
Sag-T_2_WI-Fs	3435/105.0	3/0.5	24×24	256×224	–	–
Cor-T_2_WI-Fs	3480/105.0	3/0.5	20×20	256×192	–	–
Ax-T2WI-Fs	3426/105.0	3/0.5	24×24	256×224	–	–
Ax-T2WI	3231/105.0	3/0.5	24×24	256×224	–	–
Ax-T1WI	767/9.6	3/0.5	24×24	256×224	–	–
Ax-DWI	3500/73.0	3.5/1.0	32×32	128×128	0,1000	–
Ax-DKI	3800/87.4	3.0/0.5	28×28	128×128	0,1000,2000	15
Ax-IVIM	3500/87.5	3.6/0.5	28×28	128×128	0,25,50,75,100,150,200, 400,800,1200,2000	–

DWI, diffusion weighted imaging; DKI, diffusion kurtosis imaging; IVIM, intravoxel incoherent motion.

### IVIM, stretched exponential model and DKI models

IVIM model and its parameters of diffusion coefficient (D), pseudo-diffusion coefficient (D*), and perfusion fraction (*f*) are fit for a biexponential equation:


$$S_{b}/S_{0}=\left(1-f\right)\cdot \exp\left(-b\cdot D\right)+f\cdot \text{exp}\left(-b\cdot D^{*}\right)$$

Where D characterizes extravascular diffusion of water, D* represents signal changes attributing to the intravascular movement of water, *f* is the perfusion fraction. S_b_ is the DWI signal intensity at a specified b value, and S_0_ is the baseline signal at b = 0 s/mm^2^ (20).

Stretched exponential model and its parameters of distribution diffusion coefficient (DDC) and heterogeneity index (α) are fit by the following equation:


$$S_{b}=S_{0}\cdot \text{exp}{\left(-b\cdot DDC\right)}^{\text{α}}$$

Where S_b_ represents the signal intensity at a specified b value, S_0_ is the signal intensity based on b = 0 s/mm^2^. DDC is a measure of the rate of signal decay with various b values, representing mean intravoxel diffusion rates. The heterogeneity index (α) is the water molecular diffusion heterogeneity and related to intravoxel water diffusion heterogeneity (range, 0–1). A higher α value indicates low intravoxel diffusion heterogeneity, which approaches pure monoexponential decay. Conversely, an alpha = 0 indicates a higher degree of multiexponential signal decay (21).

The DKI model is based on the following equation:


$$S_{b}=S_{0}\cdot \text{exp}\left(-b\cdot MD+\frac{1}{6}\cdot b^{2}\cdot MD^{2}\cdot MK\right)$$

Where S_b_ represents the signal intensity at a specified b value, S_0_ is the signal intensity based on b = 0 s/mm^2^. When S_0_ is known, mean diffusion (MD) and mean kurtosis (MK) are obtained. The parameter MK represents the apparent diffusional kurtosis, and MD is the diffusion coefficient that is corrected to account for the observed non-Gaussian behavior.

### Imaging and quantitative data analysis

According to PI-RADS version 2.1 (3), we screened the PI-RADS 3 lesions strictly located in TZ (**Table 2**). All the images were independently evaluated by two radiologists (JW. L., J.B.) with more than 10 years of experience in abdominal imaging diagnosis without knowing the clinical history, laboratory examination results, and other imaging examination results (such as ultrasound). In case of deviation in the results, an agreement was reached. (After PI-RADS Version 2.1 published in March 2019, we reevaluated prostate MRI of 113 patients between December 2018 and April 2019 according to PI-RADS Version 2.1)

**Table 2:** Assessment criteria for transition zone PI-RADS category 3 lesions according to PI-RADS version 2.1.

PI-RADS	Imaging assessment criteria
PI-RADS 3	1. Category 2 on T2WI (A mostly encapsulated nodule OR a homogeneous circumscribed nodule without encapsulation OR a homogeneous mildly hypointense area between nodules) and Category ≥4 on DWI (Focal markedly hypointense on ADC and markedly hyperintense on high b-value DWI)
	2. Category 3 on T2WI (Heterogeneous signal intensity with obscured margins) and Category ≤4 on DWI (Any manifestation on DWI and ADC, except that diameter of lesion ≥1.5cm in greatest dimension or definite extraprostatic extension/invasive behavior)

ADC, apparent diffusion coefficient; DWI, diffusion-weighted imaging.

**Figure 2:**
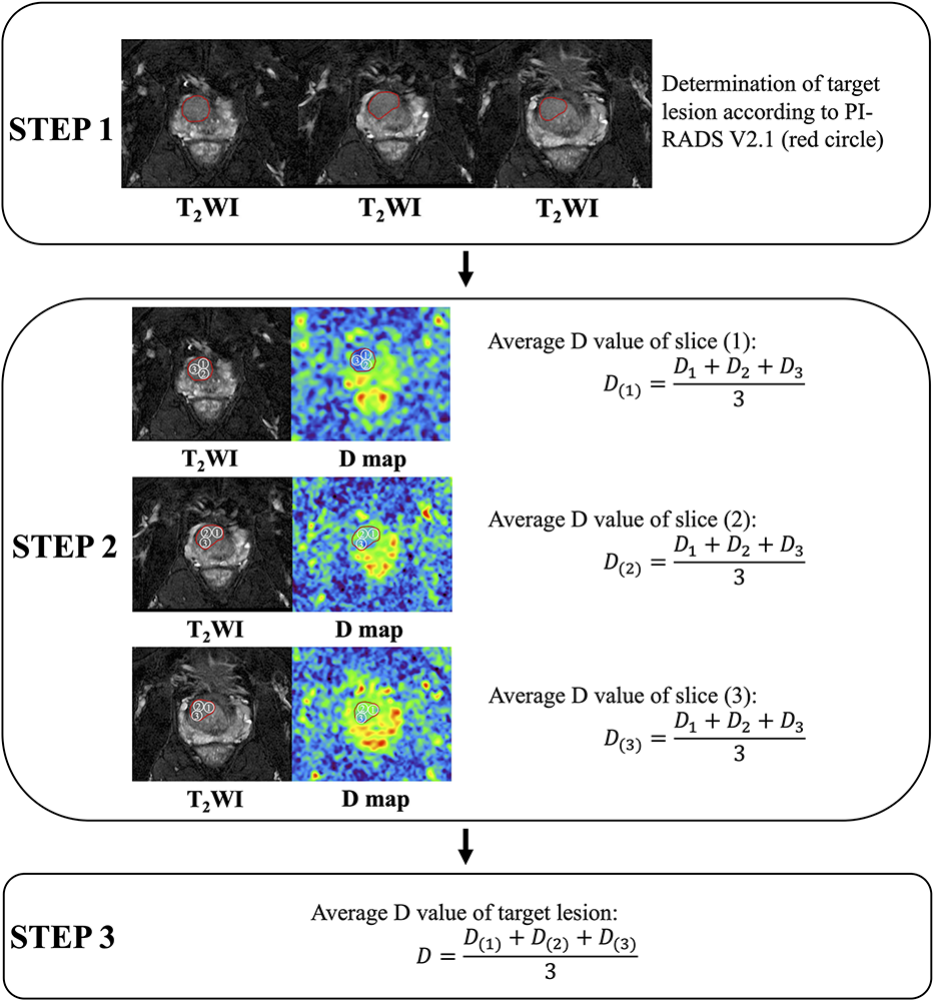
Acquisition of quantitative data (carried out on GE AW 4.6 workstation, America, GE Healthcare). Take acquisition of the diffusion coefficient (D) value as an example. Step 1. Evaluation of target lesions according to PI-RADS Version 2.1. Step 2. At the maximum 3 slices of the target lesion, measure and calculate the average value of D at each slice. Step 3. Calculating the average D value of the lesion.

Acquisition of quantitative data was carried out on GE AW 4.6 workstation. The two radiologists (KP. Z., JW. L.) depicted every region of interest (ROI) separately on related parameter maps of IVIM, stretched exponential model, and DKI. Nine ROIs with area of 50 mm^2^ were randomly drawn at the maximum 3 slices of each PI-RADS 3 lesion in TZ. The average value was recorded and used for data analysis. Detail showed in **Figure 2**.

### Biopsy

**Figure 3:**
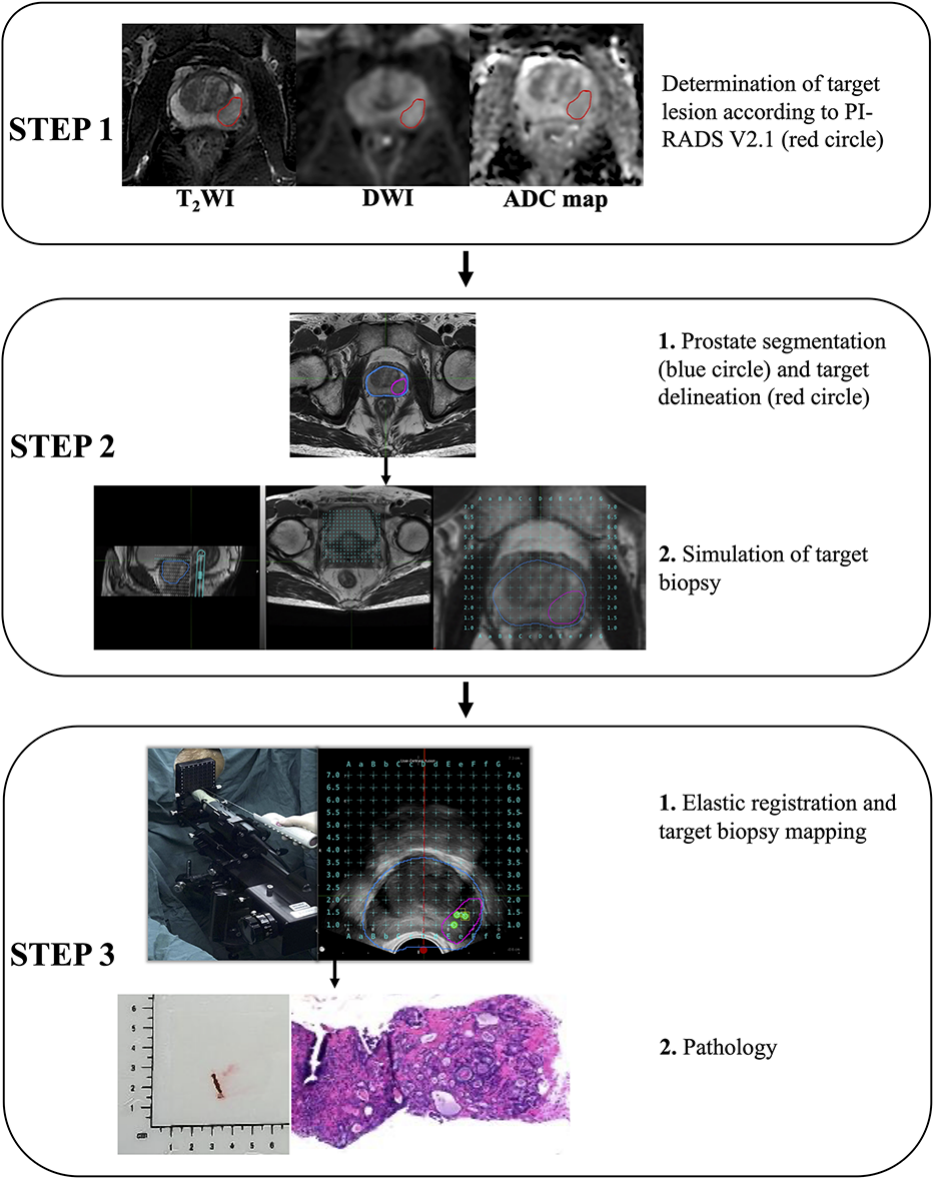
Acquisition pathology of target lesion by magnetic resonance imaging-Transrectal ultrasound (MRI-TRUS) fusion biopsy (BK Medical, Denmark).

The BK transperineal MRI/TRUS fusion biopsy system (BK Medical, Denmark) was used for all biopsies. Three target biopsy cores were taken from each target lesion before the systematic biopsies (**Figure 3**). According to the Ginsburg protocol, all patients had 18-24 systematic biopsies taken using a spring-loaded biopsy gun with an 18 gauge needle. During the systematic biopsy, 2 biopsy cores were sampled from each of 12 sectors, starting with the anterior sectors. All procedures were undertaken by 1 of 2 urologists with more than 10 years of experience in transperineal biopsy using the BK MRI/TRUS fusion biopsy system.

### Pathological analysis

All the pathological samples were separately evaluated by two pathologists (Y. L., K. G.) who were blinded to MR imaging results. In case of deviation in the results, an agreement was reached. The criteria for pathological evaluation were based on ISUP recommendations in 2014 (22). The pathological type and Gleason score of each specimen were given separately.

### Statistical analysis

One-way ANOVA was used to test whether the clinical characteristics of patients were statistically significant. In order to examine the association between parameters of DKI, IVIM, and stretched exponential model with the probability of PCa in PI-RADS 3 lesions, binary logistic regression analysis was applied. After binary logistic regression analysis, the receiver operating characteristic (ROC) curve of combination parameters, which were statistically significant in binary logistic regression analysis, were analyzed to test the diagnostic efficiency of detecting PCa in PI-RADS 3 lesions. One-way ANOVA was used to analyze whether parameters of DKI, IVIM, and stretched exponential model was helpful in differentiating BPH, PCa, and csPCa in PI-RADS 3 lesions. A *p*-value< 0.05 was considered statistically significant. All analyses were performed with SPSS (IBM, SPSS version 25).

## Results

**Figure 4:**
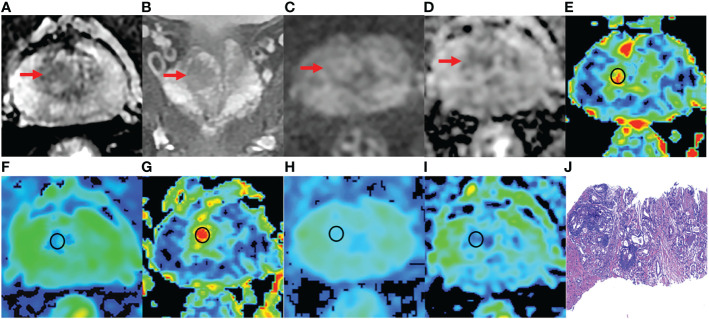
A 76-year-old man with BPH and PSA of 4.48 ng/ml. **(A-D)** The lesion with PI-RADS 3 seen in the right middle transition zone. **(E)** Fractional anisotropy (FA) of 0.201. **(F)** Mean diffusion (MD) of 1.214×10^-3^mm^2^/s. **(G)** Mean kurtosis (MK) of 0.512. **(H)** Diffusion coefficient (D) of 0.795×10^-3^mm^2^/s. **(I)** Distribute diffusion coefficient (DDC) of 0.942×10^-3^mm^2^/s. **(J)** HE staining, ×100.

**Figure 5:**
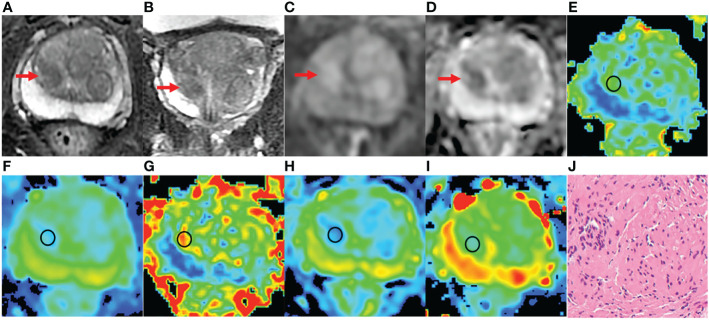
An 81 years old man with PCa, PSA of 6.47 ng/ml, and Gleason 3 + 3. **(A-D)** The lesion with PI-RADS 3 seen in the right middle transition zone. **(E)** Fractional anisotropy (FA) of 0.257. **(F)** Mean diffusion (MD) of 1.018×10^-3^mm^2^/s. **(G)** Mean kurtosis (MK) of 0.754. **(H)** Diffusion coefficient (D) of 0.588×10^-3^mm^2^/s. **(I)** Distribute diffusion coefficient (DDC) of 0.717×10^-3^mm^2^/s. **(J)** HE staining, ×100.

Among 198 PI-RADS category 3 lesions, 75.25% (149/198) lesions were pathologically confirmed as BPH (**Figure 4**), and 24.75% (49/198) lesions were PCa (**Figure 5**). In 49 PCa lesions, 37 lesions were non-csPCa (Gleason 3 + 3) and 12 lesions were csPCa (Gleason ≥ 3 + 4). Of the 145 patients with PI-RADS category 3 lesions, 68.28% (99/145) were BPH and 31.72% (46/145) were PCa. Moreover, there were 3 and each patients with 2 PCa lesions located in TZ. The average age of BPH patients and PCa patients were 73.3 years and 74.8 years (*p >*0.05), respectively. The average T-PSA of BPH patients and PCa patients was 6.17 ng/ml and 8.21 ng/ml (*p >*0.05), respectively. The detailed clinical characteristics are shown in **Table 3**.

**Table 3:** Clinical characteristics of the patients and lesions.

Variables			*p*
patients	BPH (n=99)	PCa (n=46)	
Median Age (year)	73.3(65-81)	74.8(62-87)	0.937
Median T-PSA (ng/ml)	6.17(3.25-9.88)	8.21(4.57-13.69)	0.105
Median F-PSA (ng/ml)	1.54(0.97-2.08)	1.71(1.05-4.28)	0.741
Median F-PSA/T-PSA	0.25	0.21	0.206
Median Prostate Volume (cm^3^)	69.23(49.16-81.48)	67.41(52.59-84.87)	0.698
Median PSA Density (ng/ml/cm^3^)	0.089	0.122	0.097
lesions	BPH (n=149)	PCa (n=49)	
Gleason Score		Gleason 3+3 (n=37) Gleason 3+4 (n=6) Gleason 4+3 (n=4) Gleason 4+4 (n=2)	

PSA, prostate specific antigen.

### Results of binary logistic regression analysis for predict PCa among PI-RADS 3 lesions

**Figure 6:**
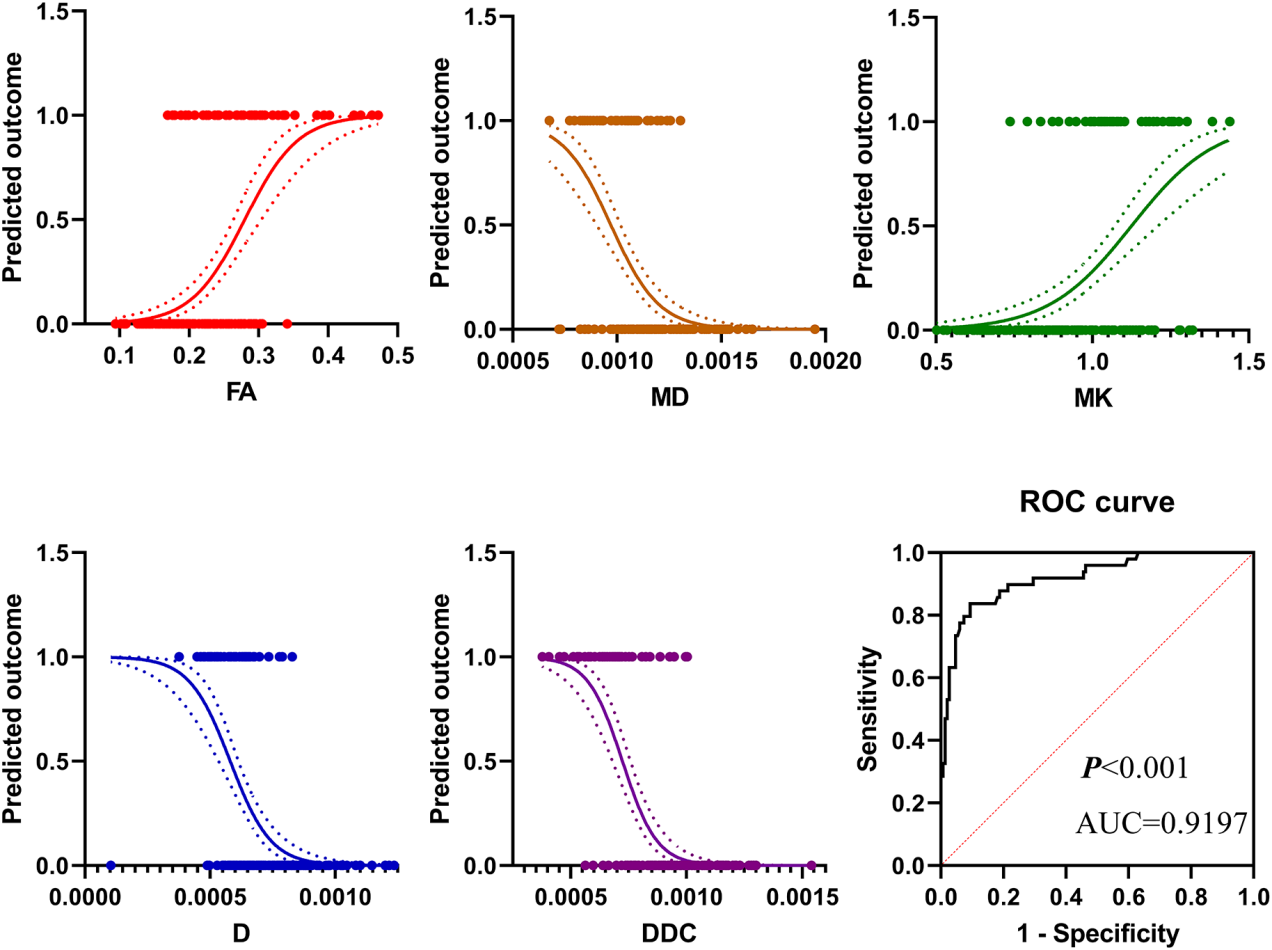
The results of binary logistic regression analysis showed that in pi-rads 3 lesions, the prevalence of PCa increased with the increase of FA value and MK value but decreased with the increase of MD value, D value, and DDC value. The diagnostic efficiency in distinguishing PCa from PI-RADS 3 lesions of these parameters was 0.9197.

Parameters fractional anisotropy (FA), MK, MD of DKI, ADC, D, D*, *f* of IVIM, DDC, and α of the stretched exponential model were included in the statistical analysis. Finally, the logistic model was statistically significant, χ2 = 181.410, *p*<0.001. The model could correctly classify 89.39% of the subjects. Among the nine independent variables included in the model, only FA (*p=0.004*), MD (*p=0.005*), MK (*p=0.015*), D (*p=0.001*), DDC (*p=0.038*) were statistically significant. Odds ratio (CI 95%) were 3.165 (2.169-8.645), 0.158 (0.008-0.427), 2.886 (1.365-7.491), 0.185 (0.019-0.382), 0.221 (0.053-0.917), respectively (**Table 4**, **Figure 6**). The results of ROC analysis showed that area under the curve (AUC) was 0.9197, CI 95%: 0.8736-0.9659. Sensitivity, specificity, positive predictive value and negative predictive value were 92.1%, 80.4%, 93.9% and 75.5%, respectively.

**Table 4:** Prediction for PCa among PI-RADS 3 lesions.

Variables	*p* Value	Odds Ratio (CI 95%)
FA	0.004	3.165(2.169-8.645)
MK	0.015	2.886(1.365-7.491)
MD	0.005	0.158(0.008-0.427)
D	0.001	0.185(0.019-0.382)
DDC	0.038	0.221(0.053-0.917)

FA, fractional anisotropy; MK, mean kurtosis; MD, mean diffusion; D, pure diffusion coefficient; DDC, distributed diffusion coefficient.

### Results of one-way ANOVA analysis

Results of one-way ANOVA analysis showed that parameters FA, MD, MK, ADC, D, DDC were statistically significant (**Table 5**). The mean value of FA and MK of csPCa (0.370 ± 0.073, 0.766 ± 0.132) were significantly higher than those of non-csPCa (0.265 ± 0.554, 0.646 ± 0.136) and BPH (0.198 ± 0.051, 0.469 ± 0.169). On the contrary, mean values of MD, ADC, D, and DDC of csPCa were significantly lower than those of non-csPCa and BPH. Compared with non-csPCa and BPH, mean values of MD, ADC, D and DDC of csPCa decreased by 0.132×10^-3^mm/s, 0.123×10^-3^mm/s, 0.063×10^-3^mm/s, 0.173×10^-3^mm/s and 0.330×10^-3^mm/s, 0.328×10^-3^mm/s, 0.243×10^-3^mm/s, and 0.434×10^-3^mm/s, respectively.

**Table 5:** Comparison of parameters between BPH, non-csPCa and csPCa in TZ PI-RADS 3 lesions.

Variables	BPH	non-csPCa (Gleason 3+3)	csPCa (Gleason≥3+4)	F	*p* value
FA	0.198±0.051	0.265±0.554	0.370±0.073	72.495	<0.001
MK	0.469±0.169	0.646±0.136	0.766±0.132	32.508	<0.001
MD (×10^-3^mm/s)	1.219±0.135	1.021±0.127	0.889±0.182	36.277	<0.001
ADC (×10^-3^mm/s)	0.883±0.118	0.678±0.122	0.555±0.117	77.584	<0.001
D (×10^-3^mm/s)	0.778±0.074	0.598±0.099	0.535±0.157	34.587	<0.001
DDC (×10^-3^mm/s)	0.965±0.105	0.704±0.131	0.531±0.160	78.382	<0.001

FA, fractional anisotropy; MK, mean kurtosis; MD, mean diffusion; ADC, apparent diffusion coefficient; D, pure diffusion coefficient; DDC, distributed diffusion coefficient; BPH, benign prostatic hyperplasia; csPCa, clinically significant prostate cancer.

## Discussion

Our study included 198 PI-RADS category 3 lesions located in TZ, where the detection rate of non-csPCa was 18.69%, and the detection rate of csPCa was 6.06%. In our study population, 23.45% of patients were non-csPCa, and thus required active surveillance at the time of initial biopsy according to NCCN guidelines. In addition, we detected 8.28% of csPCa requiring active treatment. Hansen et al. (15) found that 45.88% were PCa in PI-RADS 3 lesions located in TZ, and 21.18% were csPCa, resulting in a higher detection rate compared to the one detected in the present study. This may be because their study population was a mixed population, including the initial biopsy and the second-biopsy populations.

Typically, PCa in TZ has homogeneous low T2 signal intensity, ill-defined margins, lack of capsule, lenticular shape, and is more prone to invasion in the anterior fibromuscular stroma (1). Usually, lesions with these manifestations are assessed as PI-RADS category 4 or 5. However, in PI-RADS 3 lesions, detection of PCa using conventional multiparameter prostate MRI can be challenging. Our results showed that combining quantitative parameters FA, MD, and MK of DKI, parameter D of IVIM, and parameter DDC of the stretched exponential model could effectively identify PCa from PI-RADS 3 lesions. D value, DDC value and MD value of PCa (0.583×10^-3^mm/s, 0.662×10^-3^mm/s, 0.989×10^-3^mm/s) were significantly lower than that of BPH (0.778×10^-3^mm/s, 0.965×10^-3^mm/s, 1.219×10^-3^mm/s), which is consistent with the results of previous studies (23–25). D, DDC, and MD all reflect the diffusion of water molecules in tissues. Results of our study may be attributed to the difference in histological changes of PCa and BPH, including vascular (i.e., capillaries), fibromuscular stroma, epithelium, and glandular lumen. Compared with BPH, PCa contains increased volumes of low- restriction diffusivity epithelial cells and decreased high-restriction diffusivity stroma and lumen space. In fact, the diffusion motion of water molecules in tissues is non-Gaussian (26). In our research, FA and MK could identify PCa from PI-RADS 3 lesions. Moreover, compared with BPH (0.198, 0.469), PCa (0.291, 0.675) have higher FA and MK values. Our results are also consistent with previous studies (18, 26–28). Although it is difficult to distinguish PCa from BPH nodules by morphological features, PCa has a more complex microstructure than BPH, such as more obvious cell atypia, higher cell density, and neovascularization. These changes make the FA and MK values of PCa higher than those of PBH. Several studies have also found that a low ADC value can predict PCa in PI-RADS 3 lesions (13, 15). However, our results showed that the ADC value was not significant in our model. A plausible explanation for this may be that ADC value does not reflect the true diffusion of tissue but a combination of restricted diffusion and perfusion of tissue.

For TZ PI-RADS 3 lesions, our diagnostic model, built with parameters FA, MK, MD, D, and DDC, showed that AUC for detection of PCa was 0.9197. Sensitivity, specificity, positive predictive value and negative predictive value were 92.1%, 80.4%, 93.9% and 75.5%, respectively. Felker et al. (29) examined both clinical and MRI characteristics of 90 men with TZ PI-RADS 3 lesions, and they concluded that a combination of PSA density >0.15ng/mL^2^ and ADC value of<1000 mm^2^/sec yielded an AUC of 0.91 for csPCa. Moreover, Hermie et al. (13) showed that a combination of prostate volume<44 cc and a ratio of ADC tumor/ADC of the contralateral prostate< 70% had an AUC of 0.780 for scPCa diagnosis in PZ PI-RADS 3 lesions. Hectors et al. (30) examined T2WI radiomics features of 240 patients with PI-RADS 3 lesions and yielded an AUC of 0.76 for prediction of csPCa. In addition, both Hermie et al. and Hectors et al. wanted to predict csPCa in PI-RADS 3 lesions. However, we aimed to predict PCa in TZ PI-RADS 3 lesions, which may explain the differing results.

Active treatment is needed for csPCa (31). Screening csPCa from PI-RADS 3 lesions may be the most important task. Our research showed that parameters FA and MK of csPCa were significantly higher than those of non-csPCa and BPH. On the contrary, compared with non-csPCa and BPH, csPCa had lower MD, ADC, D, and DDC values. Park et al. (32) also found that csPCa had a higher MK value than non-csPCa and BPH, as well as a lower MD value. This undoubtedly indicated that csPCa had a more complex microstructure compared to non-csPCa and BPH. Chatterjee et al. (33) showed that the microstructure of PCA, such as gland component volumes of epithelium, stroma, and lumen space, were significantly correlated with Gleason. Also, these factors increased the degree of restriction diffusion of water molecules in tissues. Moreover, the diffusion restriction of water molecules deviating from normal distribution was more significant. As a result, the FA and MK values of csPCa increased. Several studies have found that csPCa has a lower D value and DDC value than non-csPCA and BPH. Moreover, D and DDC showed a negative relationship with the Gleason pattern (19, 25, 34), which is in line with our results. These findings strongly suggest that parameters FA, MD, MK of DKI, parameter ADC, D of IVIM, and parameter D of the stretched exponential model could be potentially used to detect csPCa.

Our results revealed that D* and *f* had no statistical significance among BPH, non-csPCa, and csPCa. On the contrary, Beyhan et al. (35) studied 31 patients, revealing that *f* of PCa in TZ was significantly lower than that of BPH. In addition, D* of PCa in TZ was also significantly higher than that of BPH, which might be because their study population was smaller than ours. Moreover, Oto et al. (36) have found that BPH nodules, dominated by stromal hyperplasia, can mimic PCA on T2WI and demonstrate early enhancement on DCE images. These findings suggest that D* and *f* cannot distinguish BPH, PCa, and csPCa. Our results showed that α wast no statistical significance, which is consistent with the previous studies (34, 37). On the contrary, Liu et al. (38) have found that α could differentiate PCa and BPH. In our clinical practice, we found that the signal of PCa was relatively homogeneous and rarely necrotic. In PCa tissue, the Gleason pattern is based on tissue architecture, including luminal, epithelial, and stromal components, and its change is based on their relative sizes rather than only tumor heterogeneity (39). These changes may support that α may not have the ability to distinguish BHP, PCa and csPCa.

## Limitations

The present research has some limitations. First, in our study, the diameter of the lesion was larger than 10 mm. Therefore, we do not know whether our results are applicable to lesions with a diameter that is less than 10 mm. Second, our sample size was small, especially csPCa, which only included 12 patients. Third, this was a single-center study, and the study results may be biased. In order to make the study results more robust and generalizable, multi-center and large sample studies may be needed to confirm our current results.

## Conclusion

In the summary, the relevant parameters of IVIM, stretched exponential model and DKI were used to establish the model, in which FA, MD, MK, D, and DDC can predict PCa in TZ PI-RADS 3 lesions and inform the decision-making process of whether or not to perform a biopsy. Moreover, FA, MD, MK, D, DDC, and ADC could differentiate csPCa and non-csPCa in TZ PI-RADS 3 lesions.

## Data availability statement

The raw data supporting the conclusions of this article will be made available by the authors, without undue reservation.

## Ethics statement

The studies involving human participants were reviewed and approved by the ethics committee of the second hospital of Dalian Medical university. The patients/participants provided their written informed consent to participate in this study.

## Author contributions

K-PZ, H-BH, CB: these authors contributed equally to this work and share first authorship. JB: this author is the corresponding author. Q-YL: this author is the co-corresponding author and contributed equally to this work and share corresponding authorship. All authors contributed to the article and approved the submitted version.

## Glossary

**Table:**

mpMRI	Multiparametric magnetic resonance imaging
PCa	prostate cancer
csPCa	significant prostate cancer
PI-RADS	Prostate Imaging-Reporting and Data System
BPH	benign prostatic hyperplasia
TZ	transition zone
IVIM	intravoxel incoherent motion
DKI	diffusion kurtosis imaging
PSA	prostate-specific antigen
ADC	apparent diffusion coefficient
FA	fractional anisotropy
MK	mean kurtosis
MD	mean diffusion
D	diffusion coefficient
D*	pseudodiffusion coefficient
*f*	perfusion fraction
DDC	distribute diffusion coefficient
α	heterogeneity index
